# Satellite clinics to bring pediatric cancer care closer to patients in Ethiopia: Perceived relevance, opportunities and anticipated challenges

**DOI:** 10.1371/journal.pone.0332074

**Published:** 2025-10-31

**Authors:** Diriba Fufa, Abdulkadir Mohamedsaid, Aziza Shad, Daniel Hailu, Hailu Alemu, Haileyesu Adam, Julie Broas, Mamude Dinkye, Mulugeta Yimer, Tadele Hailu, Zewdie Birhanu

**Affiliations:** 1 Department of Paediatrics and Child Health, Institute of Health, Jimma University, Jimma, Ethiopia; 2 Department of Paediatrics and Child Health, Tikur Anbessa Specailized Hospital, Addis Ababa University, Addis Ababa, Ethiopia; 3 Ellen W.P. Wasserman Chair of Pediatrics, The Herman & Walter Samuelson Children’s Hospital at Sinai, Baltimore, Maryland, United States of America; 4 The Aslan Project, Washington, DC, United States of America; 5 Department of Paediatric and Child Health, St. Paul Hospital Millennium Medical College, Addis Ababa, Ethiopia; 6 Department of Pediatrics and Child Health, College of Medicine and Health Sciences, University of Gondar, Gondar, Ethiopia; 7 Department of Health, Behaviour and Society, Institute of Health, Jimma University, Jimma, Ethiopia; Sunu Sante Consulting, SENEGAL

## Abstract

**Background:**

Ethiopia faces significant gaps in paediatric cancer care, with limited treatment centers and a shortage of trained professionals, leading to delayed diagnoses, high treatment abandonment, and mortality. Establishing Paediatric Cancer Satellite Clinics (PCSCs) in underserved regions offers a potential solution.

**Objective:**

This study explores the needs, relevance, and feasibility of the Paediatric Cancer Satellite Clinic (PCSC) model in Ethiopia, identifying resources, opportunities, and barriers to guide its development and implementation.

**Methods:**

This formative study was conducted from August to October 2023 in eight hospitals across Ethiopia, selected for establishing PCSCs. Data were collected through 19 focus group discussions (FGDs) with hospital and health system leaderships and purposively selected health professionals including physician, nurses, pharmacists and other categories. Data were analyzed using ATLAS.ti, with themes developed through iterative coding and categorization.

**Results:**

Participants consistently identified pediatric cancer as a significant community health issue, worsened by limited diagnostic and treatment facilities, leading to under diagnosis. The study revealed a strong unmet need for pediatric cancer care, with widespread support for establishing PCSCs to improve access. Participants expressed confidence in the feasibility and effectiveness of PCSCs, citing enablers such as existing adult oncology programs, patient demand, adequate staff, leadership interest, stakeholder support, and alignment with government plans. Opportunities included leadership backing, resource availability, partnerships with private labs, and community-based health insurance. However, challenges like inconsistent lab services and medical supplies, staff demotivation due to lack of risk allowances, role confusion, inadequate personal protective equipment (PPE), poor infection control, and insufficient psychosocial support were identified as potential barriers.

**Conclusions:**

This study underscored the urgent need for pediatric cancer care and strong support for PCSCs as a feasible and appropriate solution. Although challenges like supply chain disruptions, staff demotivation, unclear role delineation, diagnostic limitations, safety concerns, and gaps in psychosocial support-, the strong commitment of healthcare providers offer a solid foundation for success. To ensure the long-term effectiveness and sustainability of PCSCs, these obstacles must be addressed through targeted improvements in diagnostics, enhanced staff support, strengthened safety protocols, and integrated psychosocial care.

## Background

Pediatric cancer (PC) is one of the growing global health challenges. Worldwide, approximately 400,000 new cases of cancer are expected to be diagnosed in children 0–19 years of age each year [1], with the most common types including leukemia, brain tumors, and lymphomas. Despite advances in treatment, significant disparities exist between high-income countries (HICs) and low- and middle-income countries (LMICs) in terms of burden of this diseases and access to lifesaving treatment. In high-income settings, 80% or more of children diagnosed with cancer survive for at least five years. However, in LMICs, the survival rate is much lower, often below 30%, due to challenges such as late diagnosis, limited access to effective treatment and care, and inadequate healthcare infrastructure [2].

Africa carries a disproportionately high burden of PC, accounting for approximately 90,000 new cases annually [3]. Many African children present with late-stage disease, which significantly reduces their chance of treatment success and survival. The underdevelopment of healthcare systems combined with the lack of well trained professionals in many African settings leads to delayed and ineffective diagnosis and poor access to effective treatment options, making the mortality rate for PC in Africa \alarmingly high, with over 50% of affected children succumbing to this disease, compared to less than 20% in HICs [4]. Most importantly, in Sub-Saharan Africa (SSA), the majority of PC goes undiagnosed or misdiagnosed due to limited awareness from community and parents, and lacking and/or poor diagnostic facilities and capacities.

Like many other LMICs, Ethiopia experiences the lack of PC diagnostic facilities as well as a shortage of trained pediatric hematologist-oncologists and specialized cancer treatment centers, making PC one of the country’s most pressing public health and health system challenges. For instance, while the actual burden of PC in Ethiopia is estimated to be 8000−10,000 new cases annually, only a fraction of these children present to cancer treatment centers for treatment and care [5,6]. For instance, there are only five pediatric oncology units (“POUs”) available to serve Ethiopia’s 120 million person population, all of which are located at main cities in tertiary hospitals (namely, Addis Ababa, Gondar, Mekele, and Jimma). As most families live outside those major cities, for their children to access treatment, the family must travel to a PC center, which will forces them to incur significant – and in most cases insurmountable – financial hardships such as high out-of-pocket expenses for treatment, travel, and accommodation, not to mention the loss of parental income and the resultant disruptions to family life [7]. Cultural beliefs and misinformation about cancer further delay diagnosis and treatment, as many families may turn to traditional healers. or ignore early warning signs of cancer [7]. These financial and other burdens jeopardize adherence to treatment with negative psychological and social impacts. Due to a combination of these factors, mainly driven by inaccessible and distance, about 34% of Ethiopian children who start cancer treatment abandon it [8]. Moreover, the lack of a trained workforce further constrains the access to PC care in Ethiopia, with only 15 certified pediatric hematologist-oncologists and twelve fellows serving the country as of 2024.

To address the gaps in cancer care, including PC, Ethiopia developed the National Childhood and Adolescent Cancer Control Plan (NCACCP) (2019–2023). This plan outlines key strategic intervention areas such as early detection, improving diagnostic and treatment facilities, enhancing access to care, strengthening supportive services, and building human resource capacity. In alignment with this national effort, effective strategies are needed to bring pediatric cancer care closer to patients and their families [5].

One such approach is establishing satellite clinics in regions with high pediatric cancer incidence, which could significantly improve care accessibility [9]. Guided by implementation science methods, Ethiopia’s five PC treatment centers have collaboratively designed and initiated the Pediatric Cancer Satellite Clinic (PCSC) Program. The aim is to launch satellite clinics in eight remote hospitals across the country to increase access to PC treatment and reduce treatment abandonment due to the geographic inaccessibility of central cancer centers. The PCSC model focuses on mentoring and building the capacity of healthcare providers—pediatricians, pharmacists, and nurses—in these remote hospitals. Under the guidance of the main cancer centers, these healthcare professionals will manage follow-up care and treatment for children with cancer, ensuring continuity of care. Once capacity development and mentorship are established, the PCSCs will be equipped to provide chemotherapy administration, supportive care (including pain management), clinical follow-up, and referral services when necessary.

Prior to launching the clinics, the investigators of the PCSC Program recognized the need to assess the local contextual factors that would influence the success of the initiative. Accordingly, a formative study was conducted to explore the perceived burden of PC on the local healthcare systems, assess the relevance, appropriateness, and feasibility of the PCSC model, and identify existing resources, potential opportunities, and anticipated barriers. The study’s objective was to determine how the PCSC Program could both leverage existing resources and opportunities and address challenges in a systematic and planned manner in order to improve the successful implementation of the PCSCs.

## Methods

### Study setting and population

This formative study was conducted from August to October 2023 in eight hospitals that agreed to establish the planned PCSC. These hospitals included: Mattu Karl Comprehensive Specialized Hospital, Ambo University Comprehensive Referral Hospital, Bonga Comprehensive Gebre Tsadiq Shawo Memorial Hospital, Wollega University Comprehensive Referral Hospital, Hawassa University Comprehensive Specialized Hospital, Haramaya University Hiwot Fana Comprehensive Specialized Hospital, Dessie Comprehensive Referral Hospital, and Felege Hiwot Comprehensive Referral Hospital. The selection of hospitals was guided by several key criteria, including their location in underserved regions, the size and need of their catchment population, and their proximity to main cancer centers to facilitate mentorship and technical support. Additionally, the willingness and readiness of each hospital to establish a PCSC were carefully considered from the outset. This was essential, as establishing a PCSC requires not only basic infrastructure but also a strong institutional commitment to allocate the necessary resources and sustain the service over time. All but two (Bonga and Mattu) are university hospitals providing training for health professionals at undergraduate and postgraduate levels in addition to patient care. Each hospital offers comprehensive services across various departments, including inpatient and outpatient care, pediatrics, internal medicine, obstetrics and gynecology, emergency services, chronic disease management, and maternal and child health services.

Study participants were selected using a purposive sampling approach, considering individuals with relevant expertise and experience to provide richer and more insightful data. The predefined inclusion criteria focused on: (1) nurses with substantial experience in child health clinics or formal training as pediatric nurses; (2) pediatricians or general practitioners actively engaged in child healthcare services at the selected hospitals; and (3) hospital leaders, such as department heads (e.g., pharmacy, laboratory), with demonstrated leadership experience and their leadership roles and their ability to provide insights into PC care, gaps, needs, and perspectives regarding the planned PCSCs. The rationale behind this selection was to capture diverse perspectives from frontline providers and decision-makers involved in pediatric care.

Two focus group discussions (FGDs) were held at each hospital, totaling 16 FGDs, and consisting of separate groups of hospital leadership (n = 8) and technical staff (n = 8). In the leadership group, participants included hospital chief executives, medical directors, human resource directors, department heads (pharmacy, nursing, laboratory), quality control officers, and monitoring and evaluation team leaders. The technical group consisted of health professionals directly involved in patient care, including pediatricians, clinical and pediatric nurses, pharmacists, and laboratory technologists/technicians. In hospitals providing adult oncology services, health professionals engaged in adult oncology, including physicians, pediatric nurses, and pharmacists, also participated.

In addition to hospital-based data collection, focused group discussions were held at the regional health bureau (two FGDs) and one at the federal level, involving key personnel from the Ethiopian Federal Ministry of Health and representatives related organizations including the Ethiopian drug supply service (EPSS) and the national blood bank service. Each of these discussions had four to seven participants, except for those at the regional and Ministry of Health levels.

### Data collection process and tools

An open-ended discussion and interview guide [10], tailored separately for leaders and technical staff—was developed based on relevant literature and pediatric oncology guidelines [11], and used to facilitate the discussions and interviews with participants. The tool was structured according to the study objectives and covered a wide range of topics, including: experiences, needs, and burdens of pediatric cancer care; perceptions of the relevance, importance, and appropriateness of the planned clinic; potential opportunities; available resources; and barriers to implementation. The guide was reviewed and refined by pediatric hematologist-oncologists and a research methodology expert. The interviews and discussions were conducted by senior researchers with substantial experience in qualitative research. Specifically, each session was facilitated by a team of two experts: one was a public health specialist with formal training in qualitative interviewing and expertise in implementation science, and the other was a senior physician specializing in pediatric oncology. This interdisciplinary pairing ensured both methodological rigor and deep subject matter expertise. FGDs lasted between 60 and 120 minutes, while interviews ranged from 40 to 65 minutes. All sessions were recorded using a digital voice recorder in addition to note taking. Discussions with hospital leaderships and technical staffs were conducted on-site, while meetings with other stakeholders took place at their respective offices. No individuals other than participants and data collectors were present during the interviews and discussions.

### Data analysis

Data from the FGDs were transcribed verbatim and translated into English for analysis. The accuracy of the verbatim transcripts was verified through several steps. First, each interview was transcribed immediately after the session to enhance recall and ensure accuracy. Second, members of the research team randomly reviewed selected audio recordings and cross-checked them against the transcripts to confirm consistency and correctness. In addition, back-translation was conducted on selected transcripts as a quality control measure.

The coding and analysis were conducted using ATLAS.ti version 7.5., following thematic analysis approach [12]. Investigators read and re-read the transcripts, applying open coding to develop an initial coding structure. To ensure its relevance and appropriateness, the first coder (ZB) iteratively coded selected transcripts, guided by the study objectives. A second coder (DF) reviewed and validated the emergent codes. Through iterative discussions, the research team refined and reached a consensus on the coding system and definitions, which were applied to all transcripts.

Results were organized into themes and sub-themes, illustrated by key quotes. The codes and categories that guided the analysis are provided in S1 Appendix. The results are presented in relation to the perceived burden of pediatric cancer, service needs, clinic relevance, feasibility, opportunities, and barriers.

### Quality control

To ensure the trustworthiness of the findings, data were collected by subject matter experts and experienced qualitative researchers. The relevance and appropriateness of the guides were confirmed through expert review and pre-testing of the tools. After each discussion, the team held debriefing sessions to summarize key points, guiding the subsequent discussions with improved focus and an opportunity to explore new perspectives. Facilitators’ impressions were documented for each data source, and data from different participant categories were triangulated to enhance credibility and comprehensiveness. The coding and categorization process involved two coders, with ongoing feedback from the broader research team to ensure transparency and dependability of the findings.

### Ethical considerations

This study was ethically approved by the Institutional Review Board of the Institute of Health, Jimma University (Ref. No: JUIH/IRB/556/23). All participants were informed about the study’s purpose, and verbal informed consent was obtained from each respondent. During the consent process, respondents were provided with comprehensive information about the study, including its purpose, participation procedures, their right to participate or decline, and their right to withdraw at any time without any negative consequences. Respondents were then asked to indicate their decision regarding participation. If a respondent agreed, the data collector marked “Agreed”; otherwise, “Disagreed” was marked. Fortunately, no respondents declined to participate. The verbal consents were recorded and documented by the data collectors and submitted to the research team, where they are securely stored to ensure confidentiality.

### Results participants’ profile

The Focus Group Discussions (FGD) included 18 sessions across 11 institutions, with 77 participants from various hospitals and health institutions in Ethiopia and categorized into leadership and technical staff. Of the participants, 85% were male. Physicians, including general practitioners, pediatricians, and clinical leaders, constituted 37.7%. Nurses playing crucial roles in patient care and leadership made up 32.5%. Pharmacists represented 11.7%, laboratory directors 10.4%, and health officers, responsible for quality control and evaluation, made up 7.8%. These professionals, with 1–30 years of experience in patient care and aged between 21 and 50 years, contributed to discussions aimed at improving healthcare systems and standards.

### Perceived paediatric cancer burden and needs

Participants across the study settings consistently expressed concern that PC is becoming a growing issue in their community and has emerged as a significant health concern within the local healthcare system. Pediatricians and nurses interviewed indicated that they frequently encounter children presenting with symptoms suggestive of various types of cancer. However, due to the lack of diagnostic facilities, it is challenging to confirm or estimate the true burden of PC due to potentially under diagnosed childhood cancer in the community, a condition described as “an iceberg for the burden of this disease”. As a result, healthcare providers are often forced to refer these cases to higher-level medical institutions for proper diagnosis and treatment. A pediatrician noted: *P1: “It’s evident that pediatric cancer is common though we lack solid research data to confirm this. I frequently see children suffering from conditions that seem like cancer. Unfortunately, we don’t have the services here to address it. I have been referring such cases to Jimma or other medical centers. Recently, I had to refer five children for further diagnosis and treatment. This makes the proposed initiative both timely and necessary, and we should fully support it.”*-(Transcription: P3, WU_technical team).

### High demand for pediatric cancer care and service

The study revealed a significant unmet need for PC care in local hospitals and nearby settings. One pediatrician emphasized, *“The need is very high. There are many cases. Here, we only provide supportive care until they are referred to higher-level facilities.”* (*Technical staff*). Respondents, including physicians and nurses, confirmed that many children with cancer often present at hospitals in the late or terminal stages of the disease and requiring urgent care. Participants also noted that families referred to higher health facilities, such as those in Addis Ababa, frequently decline these referrals due to financial constraints, including the inability to afford travel and medical expenses. This lack of economic capacity forces many families to make the difficult decision to return home without receiving proper care. One physician explained, *“For most patients and families, Haramaya Hospital is the last option. Parents often say, ‘I will take my child home to die and focus on caring for my other children.’” (P-10-technical staff).* Another participant shared a similar observation, stating: *“I don’t think they will travel to Jimma or other places due to financial reasons. The cost of travel and cancer treatment itself is too high for them. As a result, many will return home. Even if they begin treatment, many do not complete it, often discontinuing midway. This makes the proposed project an excellent initiative, as it could help reduce treatment abandonment caused by long-distance referrals, which is the main issue.” (P1: technical).*

### Perceived relevance and importance of the planned PCSCs

Across all settings and hospitals, there was a strong consensus that the establishment of a PCSC was both timely and crucial in addressing the growing need for accessible care for children suffering from cancer. Participants emphasized that this initiative would provide much-needed services to disadvantaged families who are unable to afford the high costs of seeking medical care in distant urban centers. By bringing the services closer to these communities, the PCSC was seen as a way to promote equity and reduce disparities in pediatric cancer care. Many also mentioned that it would also offer hope to families, encouraging them to seek timely medical intervention for their children. The participants expected that the clinic would relieve the socioeconomic and emotional burdens that many families face, while also improving treatment adherence and reducing the high rates of treatment abandonment, particularly in regions where security and travel challenges make it difficult to access care.

Participants highlighted several key benefits of the PCSC. These included its potential to correct community misconceptions about PC, fostering a more optimistic outlook by raising awareness about its curability. According to the participants, the clinic would also strengthen healthcare providers’ capacity and awareness with respect to cancer treatment, contributing to the overall development of cancer care services in the region. The initiative is seen as a vital step toward ensuring fair and equal access to PC services, especially in areas where such care has been historically unavailable. A pediatrician from one of the hospitals expressed his strong support for the initiative, stating: *“I am thrilled about this idea. Starting a child oncology service here has been my hope for a long time. I’ve seen so many disadvantaged children from poor families who would otherwise die without care. If we had this service, they could receive at least some supportive care, even if we can’t always save their lives. Working in the pediatric unit, I’ve been deeply concerned about these children. I’m very happy about the proposed initiative, and we are fully prepared to support it.” (P1: technical team).*

Other healthcare providers shared similar sentiments, expressing their excitement about the prospect of starting the PCSC despite potential challenges. They emphasized their commitment to launching the service with close support and supervision from partners, believing that this initiative will play a critical role in transforming cancer care in their region.”*We are thrilled to launch the pediatric oncology satellite clinic. Despite the potential challenges we may face, we are eager to move forward with the service, relying on your close support and supervision to ensure its success (leadership].”*

Many participants stated that the introduction of PC services is expected to challenge and change community perceptions about childhood cancer. According to them, in many areas, PC is still viewed as an incurable disease. They believed that the development of the clinic, however, would raise awareness about the potential for curing PC. For example, one Hospital CEO participant noted that this initiative could help dispel the widespread misconception that cancer is always fatal, and increase the number of children seeking treatment once the clinic is operational. In hospitals where adult oncology services have already been introduced, there was a shared sense of frustration among healthcare providers regarding the lack of such services for children, viewing their absence as an “injustice.” An adult oncology nurse from one of hospitals described the expansion of pediatric oncology as essential, noting that, *“while adult services had been established, the needs of children had been overlooked.” Hospital Nurse.* In this aspect, participants stressed that the PCSC would address the long-standing issue of treatment inequity in cancer care both in terms of access to care and treatment abandonment issues; with regard to the latter, they highlighted the alarmingly high rates of treatment abandonment among pediatric cancer patients and suggested that the clinic would significantly reduce this disparity by bringing care closer to those in need. Some participants also saw the initiative as an opportunity to improve the overall quality of healthcare services within their hospitals. In their view, the establishment of the clinic is expected to enhance access to critical resources such as blood and blood products, and promote better infection prevention practices. These improvements would benefit not only oncology patients but the broader patient population as well. One official from the Ministry of Health remarked that *“the initiative would strengthen efforts to expand access to blood bank services and other healthcare components in nearby facilities,”* further contributing to the overall improvement of healthcare infrastructure in the region.

### Perceived feasibility, appropriateness, and acceptance of PCSCs

Participants were asked to reflect on the feasibility, appropriateness, and acceptance of the proposed PCSC initiative before its implementation. In the majority of hospitals visited (6 out of 8), respondents consistently expressed confidence that PCSCs are not only feasible but also an effective and acceptable approach to improving access to pediatric cancer care. This optimism was largely attributed to the presence of existing adult oncology programs, which could serve as a foundation for building and sustaining pediatric services. However, in hospitals without any oncology programs and where the number of pediatricians was limited, participants voiced some concerns about feasibility. These hospitals recognized the need for additional resources and support to successfully implement the PCSC. Despite these concerns, the overall sentiment was that the PCSC initiative represents an appropriate and much-needed model to reduce treatment abandonment, improve adherence to treatment, and strengthen follow-up care for children with cancer. National-level stakeholders and partners echoed this confidence in the feasibility of the initiative, while also acknowledging potential challenges. The KIIs at federal and regional level emphasized the urgency of launching the clinic and addressing logistical hurdles along the way. As one participant noted, *“It will be feasible. I urge you to start your activities as soon as possible. I also want to highlight that pediatric-sized blood bags are now available, which will help reduce the misuse of blood and blood products.”* (P9: MOH-level KII). Table 1 presents key factors identified by participants that contribute to the perceived feasibility of the PCSC. These include leveraging existing adult oncology services, having an adequate mix of trained staff, utilizing current resources, strong leadership, and stakeholder support, as well as prioritizing cancer drug procurement.

**Table 1:** Perceived feasibility factors for establishing Paediatric Cancer Satellite Clinics (PCSCs) in Ethiopia.

Feasibility Factors	Illustrative Quotes	Implications for implementations
**Existing Adult Oncology Program**	“The presence of adult oncology services is a solid foundation for initiating pediatric cancer care in our hospital. We can leverage shared resources and expertise.” —Hospital Leader ” I have worked in the adult oncology ward for over five years. Three years ago, we started chemotherapy for one child with cancer because families couldn’t afford to travel to Gondar for treatment. However, there has been no capacity building for pediatric cancer care.” — P1 (BSc-Adult Oncology Nurse)	Existing adult oncology services provide a foundational structure—expertise, infrastructure, and workflow—that can be adapted to pediatric cancer care, making the establishment of PCSCs more practical and resource-efficient.
**Meeting Patient Expectations**	“In my view, it is highly feasible because it aligns with the expectations of our patients.” — P3, Technical Staff	The alignment of the proposed PCSCs with patient needs and preferences enhances perceived feasibility, as local demand and community expectations support the initiative’s relevance and urgency.
**Adequate Staff Mix to Support the PCSC**	“We have a good number of pediatricians and other healthcare workers. With proper training, it would be easy to initiate and run the pediatric cancer clinic, just like we do for adults.” — P2, Technical Staff	A balanced mix of pediatricians and healthcare professionals, though not yet specialized in pediatric oncology, provides a strong human resource base that can be upskilled to support PCSC operations effectively.
**Utilizing Existing Resources and Hospital Future Plans**	“We are committed to expanding specialty services, including cancer treatment. When I speak about feasibility, it doesn’t require major infrastructure expansion or large-scale equipment procurement.” — P9, MOH Level	The feasibility is reinforced by the availability of existing facilities and alignment with institutional growth plans, minimizing the need for major investments and signaling institutional readiness.
**Committed and Motivated Staff and Leadership**	“Establishing a satellite clinic is a major step toward addressing childhood cancer care challenges. The administration is working on an implementation plan, and our staff’s commitment to serving the community makes this initiative very feasible.” — Technical Staff	Strong commitment from hospital leadership and frontline workers provides momentum for implementation, suggesting that internal motivation is a critical enabler for the success of PCSCs.
**Strong Stakeholder Support**	“We will offer mentoring, supervision, and capacity building. We are fully available for support, and I believe the project is feasible.” — P9, MOH Level	External stakeholders, especially from the Ministry of Health, offer essential mentorship and capacity-building support, indicating that inter-institutional collaboration enhances the project’s feasibility.
**Priority for Procurement**	“It will be feasible, though not without challenges. Cancer care is a priority in our organization. We are currently procuring cancer drugs without bids because the process is too time-consuming and it’s difficult to source small quantities of drugs.” — P9, MOH Level	The prioritization of cancer care in procurement policies—even through exceptional processes—signals high-level institutional backing, facilitating access to essential drugs and resources necessary for PCSC sustainability.

### Readiness for adoption of the PCSC by the target hospitals

Perceived system, setting, and staff (individual) readiness and willingness to take on and fully implement the proposed PCSCs are important parameters in implementation effectiveness. In this formative assessment, there was strong desire and interest by hospital staff to engage, initiate, and deliver the services from the new initiative across the participating hospitals. Hospital leaders and healthcare providers were interested and motivated to engage in the PCSC initiative, perceiving the initiative to be serving humanity. A participant said, “*We are so happy to start the pediatric oncology satellite clinic. Regardless of the possible frustrations, we want to start the service with your close support and supervision.*“Another stated, “*We have a strong interest and we ready to open the satellite clinic. We can just proceed to start the satellite clinic with available human resources. We will arrange the settings. We can create very good space for the new clinic very soon.* ***P 2: _leadership-hospital***

Beyond the individual interest, hospital leaders demonstrated a strong commitment to mobilize and share existing resources within the institution to initiate the clinics and some urged that the process commence quickly. Six out of the 8 assessed hospitals already had a plan to open PC services in the near future and many participants mentioned that the initiative was just a reminder and motivation as staffs were reportedly ready to grasp and harness the opportunity. “*It is our obligation to start this service, given the opportunity created now. Our nurses would have a dedication to engage and run this service with available staff and resources”* ***P 2: Hospital Leadership***

Many suggested their institutions, staff, and leadership were ready to institutionalize and integrate the initiative into their routine health care services by incorporating the PCSC into annual planning for costing and budgeting. *“We* ***[will]*** *consider the satellite clinic in the fiscal year budget and costing”*CEO. Another participant stated, “*We can start the clinic right away.* ***P 2: hospital leadership.*** Indeed, the leadership of all included hospitals expressed a willingness and commitment to allocate necessary budget allotments for PC services as part of routine care, and would place the new services into existing health service delivery packages. Participants uniformly agreed agreement that the existing capacity, human resources, and relevant expertise in the hospitals, complemented by effective capacity building, follow up, and mentorship from senior pediatric hematologist-oncologists -- particularly during the startup phase -- would enable them to initiate and sustain the planned clinic. One of the hospital managers stated, *“I believe that having a satellite clinic will be a major step in the right direction in addressing the challenges in the care of childhood cancer. The administration will be developing an implementation plan.”* They reiterated that the technical staff‘s intrinsic drive should not be money or personal gain but addressing the community’s needs.-.*technical staff*

### Readiness for adoption of the Pediatric Cancer Satellite Clinic (PCSC) by target hospitals

This assessment revealed strong enthusiasm and commitment from hospital leaders and staff to engage in and adopt this new initiative. Across all participating hospitals, there was a clear willingness to initiate and deliver PC services, with many viewing PCSCs as a humanitarian effort that align with their core mission. One participant from FHCSH stated, *“We are so happy to start the pediatric oncology satellite clinic. Despite any potential challenges, we want to begin the service with your close support and supervision.”* Another participant shared, *“We have a strong interest and are ready to open the satellite clinic. We can proceed with the available human resources and create the necessary space for the clinic very soon.”* (P2: Hospital Leadership).

Beyond individual interest, hospital leaders demonstrated a strong commitment to mobilize resources and expedite the clinic’s initiation. Six out of the eight hospitals assessed already had plans to introduce pediatric cancer services in the near future. The PCSC initiative served as a catalyst, reminding leaders of the urgency and motivating staff to seize the opportunity. As one leader from Mattu noted, *“It is our obligation to start this service, given the opportunity now. Our nurses are dedicated and will engage with the available staff and resources.” (P2: Hospital Leadership).*

Many participants expressed their institution’s readiness to adopt a PCSC, integrate it into routine healthcare, and incorporate it into their annual budgets and planning processes. One hospital CEO stated, *“We will include the satellite clinic in our fiscal year budget and costing.”* Another participant confirmed, *“We can start the clinic right away.”* (P2: Hospital Leadership). Leaders across all hospitals expressed their commitment to allocate budgets for pediatric oncology services as part of routine care. Moreover, hospital administrators confirmed that they would adjust their service delivery models to incorporate the new clinic into existing healthcare packages. Participants agreed that the available capacity, resources, and relevant expertise within the hospitals, when supplemented with close mentorship and capacity-building efforts, would enable them to successfully launch and sustain the PCSC. A hospital manager remarked, *“Having a satellite clinic will be a major step in addressing the challenges of childhood cancer care. The administration is working on an implementation plan. Our technical staff is driven not by personal gain but by the desire to meet the community’s needs.”* (Technical staff).

### Potential opportunities for the planned PCSCs

The assessment identified several key opportunities that could be leveraged to support the successful implementation of the planned PCSCs. Participants consistently highlighted a broad range of available resources within both the community and healthcare facilities, including human resources, materials, and space, all of which could facilitate the launch of the PCSCs. Most hospitals, particularly those affiliated with universities, were reported to have an adequate mix of healthcare professionals, such as pediatricians, nurses, pharmacists, laboratory technologists, and social workers. These professionals could be mobilized to provide immediate support for the PC services. However, in hospitals not affiliated with universities, staffing was more limited, representing a potential challenge that would require further attention. Table 2 outlines the resources, facilities, and infrastructure available at the time of the assessment which present significant enablers and opportunities for the PCSC initiative, providing a foundation for operational efficiency and helping the clinics gain a competitive advantage in delivering pediatric cancer care.

**Table 2:** Potential opportunities to support PCSCs.

Opportunities	Illustrative Quotes	Implications for implementations
**Technical Staff Commitment**	“Ninety percent of our staff is highly committed. I don’t foresee any issues with staff motivation. For example, our staff volunteers for special services like cleft lip surgeries to mobilize the community and boost service demand. Our team is positive and eager to contribute to this initiative, making it very achievable!” — Technical Staff	High levels of staff commitment and voluntary community involvement indicate a strong human resource base for launching PCSCs, enhancing feasibility and community engagement.
**Existing Adult Oncology Services**	“The presence of adult oncology services provides a solid foundation for initiating pediatric oncology services. We can share experiences and resources between the two services.” — Hospital Leadership	Adult oncology services create a clinical and logistical framework that PCSCs can build upon, allowing for shared resources and smoother integration.
**Support from Government and Regional Health Bureau**	“The regional health bureau is very supportive of expanding cancer care services. They have plans to open specialty services and recruit pediatricians for us.” — Hospital Leadership	Government and regional backing ensures policy alignment, staffing, and funding support, which are critical for sustainable implementation.
**Strong and Supportive Hospital and University Leadership**	“Our leadership is extremely supportive and flexible, which is crucial for our success.” — Technical Staff “ Both Dessie and Felege Hiwot Specialized Hospitals have dedicated and committed leadership, which will contribute to the success of the planned project.” — Hospital Leadership	Effective leadership at institutional and academic levels fosters a conducive environment for launching and scaling the PCSC initiative.
**Positive University Attitude**	“The university is very supportive of our hospital expansion plans and ensures budget availability for necessary drugs.” — Hospital Leadership	University-level support reflects strategic alignment with healthcare goals and facilitates access to resources and funding for PCSCs.
**Available Physical Space**	“We have additional rooms and ongoing construction projects that will soon provide adequate space for the new clinic. Once these buildings are completed, we will have the necessary rooms for various services.” — Hospital Leadership	Availability of infrastructure, including new construction and spare rooms, reduces barriers related to space and supports clinical expansion.
**Adequate Human Resources**	“We have sufficient human resources, including nursing staff and pediatricians, as well as adequate space for the new clinic.” — Hospital Leadership	Sufficient staffing across key medical disciplines improves operational readiness and feasibility of initiating specialized services.
**Government and Hospital Plans for Oncology Services**	“The Oromia Regional Health Bureau plans to start oncology services at selected hospitals, including Mattu Hospital. This initiative aligns with their regional plans and will receive necessary support.” — Hospital Leadership	Regional oncology plans indicate political will and structured support, facilitating integration of PCSCs within broader health system reforms.
**Private Diagnostic Laboratory Services**	“Private diagnostic laboratories are available nearby, and regional laboratories also provide support. This offers additional options for patient care.” — Technical Staff	Access to private and regional diagnostic services complements hospital capacity, ensuring timely and diverse diagnostics for pediatric oncology patients.
**Availability of Cancer Drugs in Private Pharmacies**	“Although there are occasional shortages of cancer drugs at the hospital, private pharmacies often have them, albeit at a higher cost to patients.” — Technical Staff	While cost may be a barrier, private pharmacies serve as an alternative source for essential medications, improving continuity of care.
**Red Cross Pharmacy Support**	“In case of drug stock outs at the hospital, many patients obtain their medications from the Red Cross pharmacy. Collaborating with them could ensure better access to drugs for patients.” — Technical Staff	Partnerships with organizations like the Red Cross can mitigate supply chain gaps and ensure more consistent drug availability.
**Established Training and Research Activities at Satellite Clinics**	“The hospital functions as a University Referral Hospital with a well-established system for service delivery, training, and research. The University plans to develop Ambo University Hospital into a center of excellence.” — Hospital Leadership	Academic integration enhances clinical training, research, and service quality, establishing a strong platform for sustainable pediatric oncology care.
**Charity Services**	“There are charities that support patients with various needs, including potentially childhood cancer. We might leverage these services for the new clinic.” — Hospital Leadership	Charitable organizations represent untapped potential to provide financial, social, and emotional support for pediatric cancer patients and their families.
**Community-Based Health Insurance**	“Community-based health insurance helps cover patients’ medical costs, alleviating financial strain on patients and their families.”	Financial protection through community-based insurance reduces treatment-related hardship and improves access to services.
**Space Arrangement for Satellite Clinics**	“All assessed hospitals have the potential to set up spaces for the planned satellite pediatric clinic. Leadership is committed to repurposing existing rooms or utilizing new constructions to meet these needs.” — Hospital Transcriptions	Space readiness, supported by leadership commitment, accelerates the physical setup and operationalization of PCSCs.
**Priority Procurement of Cancer Drugs**	“Cancer drug procurement is prioritized at the national level, which helps expedite the process and ensures availability.” — MOH Level	National prioritization of drug procurement enhances timely availability of medications, critical for uninterrupted cancer treatment.
**Role of Hospital Board of Governance**	“The hospital board composed of community representatives and stakeholders can play a crucial role in mobilizing community support and resources.” — Hospital Leadership	Community-engaged governance supports accountability, resource mobilization, and sustained community engagement in the PCSC initiative.

### Anticipated potential challenges to initiate and sustain the planned PCSCs

This assessment has identified several potential barriers and challenges that may affect the successful and effective implementation of the planned PCSC. The following section provides a brief description of these challenges, while Table 3 summarizes them along with their potential implications for the implementation of PCSCs.

**Table 3:** Potential challenges affecting the implementation of PCSCs.

Potential Challenges	Illustrative Quotes	Implications for implementations
**Lack of Safety and Personal Protective Equipment (PPE)**	“For more than a year, they worked without any safety cabinet. I can attest to that! The issue has been resolved now because Oromia supplied us with a safety cabinet. However, they started with only basic orientation, no adequate training.” – P4, Technical Staff	The lack of consistent PPE and biosafety infrastructure exposes staff to occupational hazards and reflects limitations in institutional readiness. While some improvements have occurred, insufficient training further undermines safe care delivery.
**Absence of Hazardous Waste Disposal Systems**	“Regarding waste disposal, we just store the waste in a safety box, but I’m not sure how it’s disposed of. I have expired cancer drugs stored in my room, labeled, and packed. Since I’m the only one working in that room, I take care not to use them. I don’t know how we will dispose of them in the future.” – P4, Technical Staff	Inadequate hazardous waste management poses serious environmental and health risks. The lack of clear protocols and infrastructure may undermine safe service delivery and regulatory compliance.
**Lack of Psychosocial Support Groups**	“We do not have a well-organized social support system. We only have one person who facilitates some social issues for patients, but it’s not strong or well-organized enough.” – P6, Hospital Leadership	The absence of structured psychosocial support systems can leaves families and pediatric cancer patients without crucial emotional, social, and practical assistance, limiting holistic care.
**Poor Infection Prevention and Control (IPC) Practices**	“Our infection prevention is not encouraging. We believe opening this program will help improve the hospital’s infection prevention practices by increasing awareness among staff. We need to work on this, but I don’t foresee resistance to the idea.” – P6, Hospital Leadership	Weak IPC practices could present a significant risk, especially in immunocompromised cancer patients. However, PCSCs could serve as a catalyst for broader improvements in hospital hygiene and safety culture.
**Ineffective Communication**	“There is a communication gap between the facilities and the central and peripheral EPSS hubs. Multilateral communication is needed to address these bottlenecks.” – P9, MOH-Level	Systemic weaknesses such as poor coordination, limited human capacity, and flawed communication with supply chain actors hinder reliable drug availability and operational efficiency, threatening service continuity.

#### Absence or inconsistent/irregular availability of laboratory services and diagnostic facilities.

Across study hospitals, participants expressed concern that inconsistent or irregular availability of laboratory services and diagnostic facilities is a recurring issue, and stated that frequent interruptions in the supply of necessary reagents and consumables could affect the planned PCSC. According to participants, these disruptions often result in the unavailability of essential laboratory services, such as complete blood count (CBC) analysis, organ function tests, hematology analysis, and other critical diagnostics. A participant noted: “*A major challenge is the irregular availability of laboratory services. We frequently experience disruptions. For instance, many of our lab services have been out of operation recently.” –* ***Technical Staff.*** A participant from a different hospital echoed these concerns, stating: “*We lack basic equipment such as Bone Marrow Aspiration/Biopsy sets and a centrifuge for blood component separation in the blood bank.”*-Ambo Hospital

#### Bureaucracy and delays in the supply process by EPSA.

Participants from all hospitals expressed concerns that the irregularity of medical supplies provided by the Ethiopian Pharmaceuticals Supply Agency (EPSA) posed a significant challenge to the planned clinic operations. Key informants explained that their hospitals relied heavily on EPSA for medical supplies and drugs. However, they had encountered critical gaps due to the bureaucratic nature of the procurement process and delays in supply, even when requests were submitted on time. Many doctors and leaders involved in the study emphasized that these supply chain issues had already affected patient care, particularly cancer treatment services. One notable concern was that EPSA frequently provided fewer supplies than requested by the health facilities. Furthermore, several hospital leaders reported that EPSA branches were often unwilling to issue “stock-out” letters. Without these letters, hospitals were unable to procure necessary medical supplies through alternative means, such as direct bidding with other suppliers. Participants highlighted that it was illegal for hospitals to engage in procurement without EPSA’s clearance of stock-out, further complicating the supply chain. An additional challenge involved the supply of equipment and machines by EPSA. Although EPSA provided equipment like CBC machines free of charge, these machines operated on a closed system, meaning they could only use supplies and reagents provided by EPSA. Due to the intermittent availability of these reagents, the machines often became non-functional. One participant described the situation: *“Our laboratory machine is a pre-programmed, closed system supplied by EPSA. Currently, it’s not working—its water system has failed. More importantly, the necessary reagents for the machine are unavailable, not just in the hospital, but across the country. EPSA is supposed to supply these reagents, but they are not providing them. Additionally, all of its spare parts have exceeded their due date and must be replaced, including the water system.”* – P2, Technical Staff

The data also indicated that the maintenance of machinery supplied by EPSA was another source of frustration. The bureaucratic processes and restrictions imposed by the closed system—where maintenance could only be performed by the supplier—further limited hospitals’ ability to deliver services. One participant shared: *“We frequently experience issues with our chemistry machines. We don’t have trained personnel to perform maintenance, and we can’t calibrate the machine ourselves. As a result, it often goes out of service.”* – P1, Technical Staff

#### Lack of blood and blood components for transfusions.

Participants across all settings emphasized the critical importance of having a sufficient supply of blood and blood components for cancer care, including the planned PSCS). However, many voiced concerns about shortages of whole blood (due to insufficient blood collection) and blood components (due to a lack of screening machines), which present significant challenges to both patients and the satellite clinic initiative. In most settings, except for Jimma and Nekemte, the separation of blood components is not practiced by local blood banks. Some hospitals lack machines for screening collected blood, requiring them to transport blood to distant locations such as Adama, Addis Ababa, or Jimma for screening. This delay compromises the timely availability of blood and blood components. One participant described the situation: *“We have no access to blood components. We don’t have platelets. Even whole blood is not adequately available. If we cannot secure sufficient access to blood and blood components, it will be difficult to provide cancer care, including supportive services.”* – P1, WU Technical Staff. Another participant elaborated, *“Our problem is that we don’t have a machine to screen collected blood. We have to send it to Jimma or Adama for screening, which takes a long time and often results in a lack of blood when we need it most. We also don’t have a machine to separate blood components.”* – P2,*Technical staff*

Some health workers explained that the delay in screening can sometimes cause blood to expire before it is screened, exacerbating shortages. A participant noted, *“Sometimes, the blood expires before it’s screened because of a lack of functional screening machines or the time it takes to transport blood to other locations. On the other hand, some patients die due to the unavailability of blood transfusion services. We also face shortages in human resources, such as lab technicians and nurses in the blood bank.”* – P7, Technical Staff

Key informants at various levels also identified the challenges with EPSA’s supply chain as a critical barrier to increasing access to the machines and supplies needed for screening and blood component separation. In addition to these logistical issues, concerns were raised about a lack of skills among healthcare workers and physicians in the efficient use of blood and blood products, which contributes to shortages. One participant explained, *“The machines need a sustainable supply of reagents from EPSA, but unfortunately, there are frequent interruptions in this supply. As a country, we have imported eight component-separation machines. Physicians need to encourage blood donors, and collection sites should aim for 450 ml of blood per donation. Every clinical team should be knowledgeable about the proper indications for blood and product transfusions. We are committed to training healthcare workers on the machines and ensuring the appropriate use of blood products.”* – P9, MOH Level

#### Potential staff demotivation due to lack of risk allowance for staff involved in oncology services.

Discussions with health workers in hospitals where oncology services have been practiced revealed significant dissatisfaction among staffs involved in oncology services including staff due to absence of risk allowance payment and incentives, particularly among those involved in chemotherapy preparation and administration*.* According to many participants, risk allowance is allowed only for physicians whereas pharmacists and nurses who are sharing a greater risk in the caregiving process were disallowed both risk allowance and duty hour’s payments. Moreover, even in institutions where duty payments are provided, concerns were raised that such payments were inadequate and/or inconsistent across hospitals due to a lack of clarity in the national guidelines governing health workers’ incentives and risk/duty allowances. As one participant stated, *“I think it is due to guideline. The guideline allowance risk allowance only for physicians. But there is no allowance for nurses and pharmacists who are involved cancer care and chemo therapy preparations and administrations. So, our nurses and pharmacist are not willing to work in these units, we beg and force them to work in oncology unit. They are not willing to work in this cancer unit. No nurse has an interest and willingness to join cancer treatment in our hospital. Therefore, it is essential to address these challenges before launching this new clinic as preconditions to launch this new clinic. Otherwise, it will not be sustainable.* ***P3 −4: Technical staff***

Participants expressed that this challenge will similarly affect staff motivation and willingness to take part in the planned PCSC, especially for nurses and pharmacists. In one discussion, all participants laughed and nodded their heads (to say no), when asked, “Given the absence of risk allowance, will you participate in the planned oncology services?” As one Nurse Head explained, “*We have seen it, many staff are already witnessed it that it is high risk area with no any risk allowance. So, no one is really interested to join this clinic voluntarily*”. However, in hospitals where leadership were reportedly supportive, flexible and committed to the initiative (e.g., Mattu, Wollega), duty/risk allowance payment was not emphasized as an important challenge. As one leader stated, *“We also incentivize[ ] [adult oncology healthcare professionals] by allocating a budget for duty. We pay for two nurses, and one doctors. Similarly, we will use same method for this program. We will pay duty for the duty for the nurse and physician who are going to work in these satellite clinics.***” P6: Hospital Leadership**

### Potential staff demotivation due to lack of risk allowance for oncology staff

Discussions with healthcare workers in hospitals providing oncology services revealed significant concerns and dissatisfaction among staff, particularly those involved in chemotherapy preparation and administration, due to the absence of risk allowances and incentives. Many participants emphasized that while physicians receive risk allowances, pharmacists and nurses—who are also exposed to considerable risks in the care process—are excluded from these payments, as well as from duty hour compensation. Additionally, concerns were raised about inconsistencies in duty payments, which were reportedly inadequate and varied between hospitals. These inconsistencies were attributed to a lack of clarity in the national guidelines on health workers’ incentives and risk/duty allowances. One participant expressed frustration: *“I believe it’s due to the guidelines. Risk allowances are only provided to physicians, but there’s no allowance for nurses and pharmacists who are involved in cancer care and chemotherapy preparation and administration. As a result, our nurses and pharmacists are reluctant to work in these units. We have to beg and pressure them to stay in oncology. No nurse is interested in joining the cancer treatment unit in our hospital. It’s crucial to address these challenges before launching the new clinic. Otherwise, it won’t be sustainable.”* – P4, Technical staff

Participants consistently expressed concerns that this lack of risk allowance would continue to demotivate staff, particularly nurses and pharmacists, making them unwilling to participate in the planned Primary Satellite Cancer clinic (PCSC). In one discussion, when participants were asked whether they would be willing to take part in the planned oncology services without a risk allowance, *all* participants shook their heads in disapproval, with some even laughing in response. One nurse leader remarked: *“We’ve already seen it. Many staff members have witnessed firsthand that it’s a high-risk area with no risk allowance. No one is interested in joining this clinic voluntarily.”* – Nurse Head

However, in hospitals where leadership was supportive and flexible—such as in Mattu and Wollega—the issue of risk and duty allowances was not as pronounced. Hospital leaders in these areas had reportedly found ways to incentivize staff through additional budget allocations for duty payments. One leader explained: *“We incentivize our staff by allocating a budget for duty payments in oncology. We pay for two nurses and one doctor. We plan to follow the same approach for this program. We will provide duty payments to both the nurse and physician working in these satellite clinics.”* – P6, Hospital Leader.

#### Role conflict and confusion regarding nurses’ and pharmacists’ roles in chemotherapy drug preparation and administration.

In nearly all hospitals involved in the study, participants highlighted role conflicts and confusion between nurses and pharmacists regarding the preparation and administration of chemotherapy drugs. These inconsistencies and conflicts could potentially affect the planned PCSC, according to many participants. In some hospitals, nurses were responsible for both the preparation and administration of chemotherapy drugs, while, in others, pharmacists handled the preparation. This lack of standardization has led to confusion, role conflict, poor teamwork, and a lack of cooperation between the two groups. One participant noted, *“There is confusion and role conflict about who should prepare chemotherapy drugs. In some hospitals, this is the nurses’ responsibility, while in others, it’s the pharmacists’. Here, we don’t have clearly defined roles, and it has caused some conflict. These roles should be clearly defined and established moving forward.”* – P4, Technical Staff

Another participant raised concerns about the availability and cost of chemotherapy drugs, emphasizing the need for clear coordination with the EPSA: *“Another concern is the availability of chemotherapy drugs at our hospital. These drugs are very expensive, so there needs to be a well-organized logistics system in coordination with EPSA. Chemotherapy preparation should be done by pharmacists.”* – P3, Pharmacy Director

## Discussion

This formative study aimed to generate essential data to guide the design and development of the PCSC Program, with the ultimate goal of improving access to care for children in underserved areas. Through the perspectives of healthcare providers, the study explored the need and demand for PC treatment, while assessing the acceptability, relevance, appropriateness, feasibility, and perceived importance of the proposed PCSCs. The findings revealed that the concept of PCSC initiative received widespread recognition and acceptance, highlighting its potential to fill a critical gap in PC care. The study identified key factors influencing the perceived feasibility of the PCSCs, as well as the readiness of target hospitals to adopt the clinics. Healthcare providers consistently emphasized the growing burden of PC within the local health system. However, due to a lack of diagnostic infrastructure and trained personnel, estimating the true extent of the disease remained challenging. Healthcare workers often referred suspected cases to distant facilities for evaluation and diagnosis, but many parents were unable or unwilling to travel long distances due to economic constraints, leaving their children without essential care and their disease uncounted.

In light of these critical challenges and gaps, there was strong consensus among healthcare workers and stakeholders that the local health system urgently needed PC services. The proposed decentralization of care that would result from the PCSCs was deemed the most appropriate and relevant solution to address the significant unmet need such services in local settings. The establishment of PCSCs was not only seen as timely but crucial in addressing the growing demand for accessible cancer care. The proposed approach was praised for its potential to promote equity and reduce disparities by offering a lifeline to disadvantaged families through the provision of care closer to home. More specifically, the approach was widely commended for its potential to reduce the socioeconomic and emotional burdens that many families of children with cancer face, thereby expecting to improve treatment adherence, decrease high rates of treatment abandonment, and correct common misconceptions about childhood cancer. Raising awareness about the curability of pediatric cancer would also seen as a means to encourage a more optimistic outlook within the community and improve the overall quality of healthcare services in local hospitals.

Literatures similarly supports that decentralizing cancer treatment and care through satellite clinics in low-resource settings can significantly enhance patient outcomes by enhancing access to care, reducing travel burdens and costs to patients and families, [13] fostering early detection and community awareness [14], assisting local healthcare providers in building capacity and improving continuity and coordination, addressing both treatment and follow-up needs [15,16], and, finally, by enhancing patient trust and adherence [17–19].

With these considerations in mind, the proposed PSCS Program was enthusiastically supported by leadership at all levels, including hospitals, regional, and federal authorities. Healthcare workers and partners expressed strong interest, readiness, and motivation to adopt and launch the PCSCs according to the planned timeline, viewing it as a transformative step in addressing PC care in remote and/or underserved areas. Strong support was reflected both in terms of allocating necessary resources (human, materials, finances/budget, and administrative) and arranging the facilities and spaces to host the satellite clinics. Such widespread acceptance and support will lay a solid foundation for the clinics, ensuring smooth operations and long-term sustainability. Additionally, to ensure the long-term sustainability, it is essential to implement strategies such as fostering public-private partnerships, exploring government and international funding opportunities, transitioning to value-based care models, and integrating technology and automation. In support of these findings, earlier works underscored that, in many settings, the satellite clinic models are well-received by stakeholders and healthcare workers as a way to increase access to pediatric cancer PC care in underserved settings [20–22].

The study also documented that the planned PCSCs was widely perceived as feasible by healthcare workers, hospital staff, and national stakeholders. This perceived feasibility stems from several interconnected factors identified by participants: the potential to leverage existing adult oncology services; the presence of a well-trained mix of staff; adequate current resources; strong stakeholder support; alignment with patient expectations; and proactive logistical planning.

The reliance on existing adult oncology services to establish PC care is a significant finding that aligns with studies from similar contexts, which demonstrate consistently that leveraging existing healthcare infrastructure and resources and coordinating with future organizational service expansion plans are crucial for the successful implementation of new services, particularly in resource-constrained settings [23]. These same studies highlight that integrating PC into established adult cancer programs can significantly reduce the time, cost, and logistical challenges associated with launching entirely new initiatives. These strategies are particularly compelling in Ethiopia, where healthcare infrastructure, resources and trained human resources are unevenly distributed and concentrated in urban areas. However, while the presence of adult oncology programs is advantageous to the establishment of the planned clinics, the adaptation of services from adult to PC requires significant modifications in protocols, training, and resources as existing adult services and resources may not sufficient to meet the full scope and specific needs of PC requirements; thus, without targeted investment and resource allocations in specialized training and infrastructure, the planned PCSC Program may not be effective. The study outlined that the fact the planned clinic aligned with patient expectations enhances the feasibility of the PCSC by ensuring that the clinic addresses the actual needs of the community, which can boost acceptance and utilization of the services offered. However, it is crucial to strike a balance, as there is a risk that the clinic may face “over-expectation challenges [24].” If the planned services cannot fully meet the high or unrealistic expectations of families and patients, it could result in dissatisfaction and undermine the clinic’s effectiveness.

A well-trained staff mix is also crucial for the feasibility of pediatric cancer clinic as the presence of pediatricians and trained healthcare workers is essential for successful implementation. The present study revealed that, with exception of a few facilities, there already existed a mix of doctors and nurses committed to supporting and contributing to the planned PCSCs. Such existing resources, coupled with the equally committed leadership at all levels of the healthcare system, participants expressed confidence that the planned PCSCs would not require significant new infrastructure. This perceived feasibility factor suggests that the PCSCs can be set up using existing resources and infrastructure with targeted resource inputs, such as capacity-building efforts to equip existing healthcare workers with essential skills in PC care and patient support processes. However, even with specialized training in pediatric oncology for pediatricians and nurses, relying on existing staff will require careful evaluation. This is because PC treatment and care generally requires specific equipment and/or adjustments to facilities tailored to adult oncology as well as investments in infrastructure such as specialized wards for children that could still impact feasibility. Therefore, while building on existing resources is essential, careful planning and resource assessment and allocations are also needed.

On the other hand, the study found that commitment of staff and leadership is a critical enabler and opportunity for the PCSCs’ success as it creates a positive environment for launching the clinic. Additionally, strong stakeholder support, including external mentorship and capacity building, represents another major opportunity for the clinic’s success. The MOH and other entities appear committed to providing supervision and support, which will be critical for the clinic’s early and ongoing operations. However, the feasibility of this support will depend on consistent and timely engagement. It is one thing to have support in principle, but another to receive the required resources and mentorship consistently over time. The success of the PCSCs may hinge on the reliability of this external assistance, and any delays or gaps in support could jeopardize feasibility. This opportunity is significant because it ensures that there is buy-in from key stakeholders. However, maintaining this level of commitment long-term will require sustained efforts, particularly in the face of potential challenges such as workload increases, resource constraints, and evolving healthcare demands. Ongoing support and leadership engagement thus are crucial to keeping the clinic operational and effective.

The study also highlighted that the planned PCSCs have several additional opportunities that may enhance their feasibility and sustainability. Partnering with private diagnostic laboratories would provide access to advanced diagnostic tools, alleviating the hospitals’ diagnostic burden and improving care quality. Similarly, collaboration with private pharmacies and the Red Cross Pharmacy could enhance access to affordable cancer drugs, bypass complex procurement processes, and reduce financial barriers to care, thereby prevent treatment delays. Indeed, the existence of Community Based Health Insurance (CBHI) is another greater opportunity for panned clinic because it would plays important role to cover cancer treatment could reduce the financial burden on families and increase patient access to services. Charity organizations also present a significant opportunity, as they can provide financial support to cancer-affected families’ assist the planned PCSCs by donating medical equipment and supporting staff capacity development, and increase community awareness. Hospital leadership and governance can play a crucial role in engaging such organizations by mobilizing resources, prioritizing the clinic’s needs, and securing external support from stakeholders [25].

The assessment also revealed critical and complex challenges and barriers that could impact the development and ongoing operation of the planned PCSCs. One of the most critical challenges threatening the successful implementation of the planned PCSCs, in the current setting where resources limited, is the bureaucratic and inefficient nature of drug procurement and supply chain systems. Delays in accessing essential cancer medications and diagnostics—often due to rigid procurement procedures, limited coordination with the EPSS, and weak forecasting by healthcare workers and hospitals—result in frequent treatment interruptions. These gaps undermine patient trust and increase out-of-pocket costs for families, placing additional burdens on already vulnerable households. Furthermore, the irregular availability of laboratory services and diagnostic tools, frequently caused by supply interruptions and equipment shortages, could impair timely and accurate diagnosis. This not only delays clinical decision-making but can also lead to life-threatening consequences for children with cancer. The study also highlights that the bureaucratic procurement processes managed by EPSS directly affect the availability and maintenance of critical equipment and the timely supply of blood and blood components. Without addressing these structural inefficiencies, the functionality and credibility of the planned PCSCs remain at serious risk. Therefore, there is an urgent need to reform procurement pathways to allow for greater flexibility and prioritization of pediatric oncology supplies. Strengthening coordination between PCSCs and EPSS, implementing basic stock management tools, and building partnerships with private pharmacies, NGOs, and regional blood banks can enhance supply reliability. In addition, adopting a more decentralized or hub-and-spoke distribution model may improve the responsiveness and resilience of supply chains, ultimately ensuring continuity of care and strengthening the sustainability of PCSCs.

Another potential challenges noted by the study participants was the lack of clear roles and responsibility throughout the healthcare system, resulting in role conflict among the providers -- especially among between nurses and pharmacists in chemotherapy preparation and administration -- that could disrupt treatment and shouldn’t been seen as simple factor as may cause chaos in the coordinated service provisions. These role ambiguities and conflicts underscore the need for clearly defined responsibilities to ensure smooth collaboration, communication, and positive team spirit among healthcare providers that are critical to the successful implementation of the PCSC program. On the other hand, staff demotivation stemming from lack of risk allowances inadequate personal protective equipment, poor waste disposal system/facilities, and poor infection prevention practice systems could affect both staff morale and patient care and safety. Another critical barrier is the absence of psychosocial support at satellite clinics, which can significantly undermine the success of the planned PCSCs. Without dedicated personnel or structured support systems, families may experience emotional distress, financial hardship, and social isolation—factors that contribute to treatment non-adherence and abandonment. Additionally, the lack of support places emotional strain on healthcare workers, potentially affecting the quality of care. Therefore, it is essential that PCSCs integrate basic psychosocial services by training existing staff in supportive care, establishing peer or parent support groups, and partnering with local NGOs or community organizations to provide holistic emotional and social support.

These challenges underscore the need for effective strategies at both the co-design and planning phases as well as throughout the future operation of the satellite clinic.

### Strength and limitation of the study

This formative assessment utilized a qualitative methodology to explore the potential acceptance, feasibility, existing opportunities, and challenges surrounding the development of PCSCs in our resource-limited setting. In-depth, participatory discussions were conducted, providing valuable insights from key stakeholders, particularly physicians, pediatricians, nurses, and frontline health workers involved in direct patient care. Hospital leadership and partners at various levels were also engaged, ensuring a broad perspective. The interviews and discussions were led by a senior pediatric hematologist-oncologist alongside an expert in implementation science and qualitative research, combining subject matter expertise with methodological rigor to enhance the study’s depth and conceptualization. However, a notable limitation is the absence of patient perspectives, which could have enriched the findings. Future phases of this project should address this gap by incorporating patient voices into the assessment.

## Conclusion

This formative study, conducted in Ethiopia, aimed to expand access to childhood cancer care by establishing Pediatric Cancer Satellite Clinics (PCSCs) through capacity building and mentoring of local healthcare workers. The findings demonstrate strong overall readiness for and widespread acceptance of the proposed clinic model among healthcare providers, hospital leadership and staff, and other key stakeholders. Participants emphasized the urgent need for decentralized pediatric cancer clinic to address the increasing burden of childhood cancer, especially in underserved areas and regions. Leveraging existing healthcare infrastructure and resources—particularly adult oncology services—and mobilizing committed and health staff and leadership at various levels were identified as significant opportunities for the successful implementation of the clinics. The study highlighted the feasibility of the PCSC model, with participants recognizing its potential to improve care accessibility, reduce travel burdens on families, and enhance treatment adherence. The study underscored the readiness of and enthusiasm within Ethiopia’s health system to adopt the PCSC model, provided that logistical, resource, and training challenges are addressed. The clinics’ implementation is seen as a transformative step toward improving childhood cancer care in Ethiopia, fostering equity, and reducing disparities in access to life-saving treatments. The insights from this study provide a roadmap for the ongoing implementation and healthcare providers as they work to implement this critical initiative.

Despite the positive outlook, several challenges were noted; including limited diagnostic infrastructure, inconsistent supply chains, limitations in staff invectives and motivations, and a shortage of human resources specialized in pediatric oncology. Addressing these barriers will be crucial to ensuring the clinic’s long-term sustainability and effectiveness. Strong partnerships with private diagnostic laboratories, pharmacies, and external organizations, targeted interventions in training to build capacity, the procurement of necessary resources, and ongoing stakeholder support will also be essential to support the clinics’ success.

## Supporting information

S1 Appendix Thematic coding framework. Results were organized into overarching themes and sub-themes, supported by illustrative participant quotes. The codes and categories that guided the thematic analysis are presented in this appendix.
(DOCX)
