# Supplementary material for: Satellite clinics to bring pediatric cancer care closer to patients in Ethiopia: Perceived relevance, opportunities and anticipated challenges
Source: PLoS One. 2025 Oct 31;20(10):e0332074. doi: 10.1371/journal.pone.0332074 (PMC12578237; doi:10.1371/journal.pone.0332074)
Supplement: S1 Appendix — Results were organized into overarching themes and sub-themes, supported by illustrative participant quotes. The codes and categories that guided the thematic analysis are presented in this appendix. (DOCX) [file pone.0332074.s001.docx]

**Code-Filter: All**

______________________________________________________________________

HU: Mychild_atlas

File: [D:\Zewde file\Diriba files\Trascriptions\Mychild_atlas.hpr7]

Edited by: Super

Date/Time: 2025-06-07 22:39:20

______________________________________________________________________

**PD burden**

**PD burden and relevance of**

**PD burden and relevance of-perception**

**Access challenges: Distance**

**Access challenges: patients do not follow their referral**

**Access challenges: economy**

**Available resources: chemo safety**

**Available resources: COVID-19 PPEs**

**Available resources: dedicated space**

**Available resources: effective blood bank**

**Available resources: Functional Pediatric OPD**

**Available resources: good IPC**

**Available resources: human resources**

**Available e resources: Culture service**

**Available resources: rooms and space**

**Challenges to cancer care: no adequate training**

**Challenges to care: lack of safety equipment**

**Challenges to care: role confusions but nurse and pharmacist**

**Challenges to care: skill gaps**

**Concerns: Training 2 staffs may not be enough**

**Duty**

**Feasibility**

**Insurance**

**Need- Available resources like chemotherapy**

**Need-training-pharmacists**

**Need-training on blood screen and transfusions indications**

**Need-training psychosocial**

**Need-training: IPC**

**Need-training: laboratory**

**Need-training: onsite training**

**Need-training: physicians**

**Need-training: hospital leadership**

**Need: Functional mechanical ventilator**

**Need: human resource increase**

**Need: office furnish and materials**

**Need: Training and mentoring**

**Need: Training: nurses**

**Opportunities: adequate supplies**

**Opportunities: Adult oncology service**

**Opportunities: better drug availability**

**Opportunities: better experience in initiative maintenance**

**Opportunities: cancer drugs purchased without BID**

**Opportunities: cancer drugs sometimes available in private pharm**

**Opportunities: cataract charity**

**Opportunities: close ties with blood bank-location within hospital**

**Opportunities: committed leaders**

**Opportunities: Having pediatrician**

**Opportunities: Health insurance**

**Opportunities: linkage with JU**

**Opportunities: partner with regional lab**

**Opportunities: pathologist on training**

**Opportunities: private laboratory services in towns**

**Opportunities: quality monitoring team existence**

**Opportunities: readdress pharmacy partner**

**Opportunities: regional HB plan to start oncology**

**Opportunities: regional ORHB plan to start oncology**

**Opportunities: ST scans to be started**

**Opportunities: supportive hospital board**

**Opportunities: supportive hospital leadership**

**Opportunities: University hospital with good resource**

**Opportunities: adequate and committed staff**

**Opportunities: aligned with hospital visions**

**Opportunities: committed ad passionate staff**

**Opportunities: constructions underway**

**Opportunities: good incentive practice**

**Opportunities: good practice in duty payment**

**Opportunities: good support from ORHB**

**Opportunities: rooms and buildings under constructions**

**Opportunities: specialty service in place or ready to open**

**Opportunities: Supportive university leadership**

**Potential challenges: drug shortage**

**Potential challenges: ICU unit**

**Potential challenges: inappropriate setup for chemo preparations**

**Potential challenges: Increase demand**

**Potential challenges: lack of blood components Segregation**

**Potential challenges: lack of blood culture**

**Potential challenges: lack of cancer drugs**

**Potential challenges: Lack of cancer registry system**

**Potential challenges: lack of chemo drugs**

**Potential challenges: lack of confidence by nurses**

**Potential challenges: lack of effective social support system to patients**

**Potential challenges: lack of laboratory service**

**Potential challenges: lack of proper waste disposal system**

**Potential challenges: lack of skill on chemo preparation**

**Potential challenges: lack of some tests like electrolyte**

**Potential challenges: limited CBC machine**

**Potential challenges: low community awareness**

**Potential challenges: low staff awareness about cancer**

**Potential challenges: no capacity to screen blood at local**

**Potential challenges: no capacity to screen blood at local-lack of reagents**

**Potential challenges: no willingness among staff to join cancer care**

**Potential challenges: poor drug quantification capacity**

**Potential challenges: poor IPC**

**Potential challenges: service cost to pts**

**Potential challenges: staff attitude toward chemotherapy**

**Potential challenges: nonfunctional CBC machine**

**Potential challenges: absence of lab services**

**Potential challenges: blood bank and lack of blood components**

**Potential challenges: complicated procedures at EPSA**

**Potential challenges: complicated procedures at EPSA-poor communication**

**Potential challenges: demotivated staff-no risk allowance**

**Potential challenges: human resource increase**

**Potential challenges: irregularity of cancer drugs**

**Potential challenges: irregularity of lab services**

**Potential challenges: irregularity of lab services and supplies**

**Potential challenges: lack of basic equipment**

**Potential challenges: lack of pharmacist**

**Potential challenges: lack of psychosocial support**

**Potential challenges: No risk allowance**

**Potential challenges: poor attention to cancer care by hospital**

**Potential challenges: poor facilities like lab**

**Potential challenges: security situations**

**Potential challenge SC: interruptions of essential antibiotics**

**Potential challenge SC: lack of reliably functioning chemistry machine**

**Potential challenge SC: closed lab machines**

**Potential challenge SC: finance to support patients**

**Potential challenge SC: increase workload to physician**

**Potential challenge SC: lack of blood and blood products**

**Potential challenge SC: shortage of space**

**Potential challenges SC: lack diagnostic facility**

**Potential challenges: Functional mechanical ventilator**

**Readiness**

**Satellite clinic initiative: timely and relevant**

**Stakeholders**

**Stakeholders: community**

**Stakeholders: hospital board**

**Stakeholders: merchants and resourceful persons**

**Stakeholders: NGO**


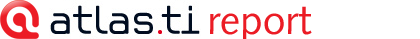


**Families: List of Code Families and their Members**

| **Code Family** | **Codes** |
| --- | --- |
| Potential challenges | - Potential challenges: drug shortage - Potential challenges: ICU unit - Potential challenges: inappropriate setup for chemo preparations - Potential challenges: Increase demand - Potential challenges: lack of blood components Segregation - Potential challenges: lack of blood culture - Potential challenges: lack of cancer drugs - Potential challenges: lack of chemo drugs - Potential challenges: lack of effective social support system to patients - Potential challenges: lack of laboratory service - Potential challenges: lack of proper waste disposal system - Potential challenges: lack of some tests like electrolyte - Potential challenges: limited CBC machine - Potential challenges: low community awareness - Potential challenges: no capacity to screen blood at local - Potential challenges: no capacity to screen blood at local-lack of reagents - Potential challenges: no willingness among staff to join cancer care - Potential challenges: poor drug quantification capacity - Potential challenges: poor IPC - Potential challenges: service cost to patients - Potential challenges: nonfunctional CBC machine - Potential challenges: absence of lab services - Potential challenges: blood bank and lack of blood components - Potential challenges: complicated procedures at EPSA - Potential challenges: complicated procedures at EPSA-poor communication - Potential challenges: demotivated staff-no risk allowance - Potential challenges: human resource increase - Potential challenges: irregularity of cancer drugs - Potential challenges: irregularity of lab services - Potential challenges: irregularity of lab services and supplies - Potential challenges: lack of basic equipment - Potential challenges: lack of pharmacist - Potential challenges: lack of psychosocial support - Potential challenges: No risk allowance - Potential challenges: poor attention to cancer care by hospital - Potential challenges: poor facilities like lab - Potential challenges: security situations - Potential challenge: interruptions of essential antibiotics - Potential challenge: lack of reliably functioning chemistry machine - Potential challenge: closed lab machines - Potential challenge: finance to support patients - Potential challenge: increase workload to physician - Potential challenge: lack of blood and blood products - Potential challenge: shortage of space - potential challenges: lack diagnostic facility - Potential challenges: Functional mechanical ventilator |
| Existing Resources | - available resources: chemo safety - available resources: COVID-19 PPEs - available resources: dedicated space - available resources: effective blood bank - available resources: Functional Pediatric OPD - available resources: good IPC - available resources: human resources - available resources: Culture service - available resources: rooms and space |
| Opportunity | - opportunities: adequate supplies - opportunities: Adult oncology service - opportunities: better drug availability - opportunities: better experience in initiative maintenance - opportunities: cancer drugs purchased without BID - opportunities: cancer drugs sometimes available in private pharm - opportunities: cataract charity - opportunities: close ties with blood bank-location within hospital - opportunities: committed leaders - opportunities: Having pediatrician - opportunities: Health insurance - opportunities: linkage with JU - opportunities: partner with regional lab - opportunities: pathologist on training - opportunities: private laboratory services in towns - opportunities: quality monitoring team existence - opportunities: readdress pharmacy partner - opportunities: regional HB plan to start oncology - opportunities: regional ORHB plan to start oncology - opportunities: ST scan to be started - opportunities: supportive hospital board - opportunities: supportive hospital leadership - opportunities: University hospital with good resource - opportunities: adequate and committed staff - opportunities: aligned with hospital visions - opportunities: committed ad passionate staff - opportunities: constructions underway - opportunities: good incentive practice - opportunities: good practice in duty payment - opportunities: good support from ORHB - opportunities: rooms and buildings under constructions - opportunities: specialty service in place or ready to open - opportunities: Supportive university leadership |
| stakeholders | - stakeholders - stakeholders: community - stakeholders: hospital board - stakeholders: merchants and resourceful persons - stakeholders:NGO |
|  |  |
| Training and capacity needs | - need-training: IPC - Need-training: laboratory - need-training: onsite training - Need-training: physicians - Need-training: hospital leadership - Need: Functional mechanical ventilator - Need: human resource increase - Need: office furnish and materials - Need: Training and mentoring - Need: Training: nurses |

| Need-available resources like chemotherapy | - Training and capacity needs |
| --- | --- |
| Need-training-pharmacists | - Training and capacity needs |
| Need-training on blood screen and transfusions indications | - Training and capacity needs |
| Need-training psychosocial | - Training and capacity needs |
| need-training: IPC | - Training and capacity needs |
| Need-training: laboratory | - Training and capacity needs |
| need-training: onsite training | - Training and capacity needs |
| Need-training: physicians | - Training and capacity needs |
| Need-training: hospital leadership | - Training and capacity needs |
| Need: Functional mechanical ventilator | - Training and capacity needs |
| Need: human resource increase | - Training and capacity needs |
| Need: office furnish and materials | - Training and capacity needs |
| Need: Training and mentoring | - Training and capacity needs |
| Need: Training: nurses | - Training and capacity needs |
| Readiness |  |
| satellite clinic initiative: timely and relevant |  |
| Stakeholders | - stakeholders |
| stakeholders: community | - stakeholders |
| stakeholders: hospital board | - stakeholders |
| stakeholders: merchants and resourceful persons | - stakeholders |
| stakeholders: NGO | - stakeholders |
